# Establishment of antiretroviral pediatric registry: efforts toward achieving the UNAIDS 95-95-95 targets in the Free State Province, South Africa

**DOI:** 10.3389/fpubh.2025.1623386

**Published:** 2025-07-30

**Authors:** Bukola R. Omotoso, Mukesh Dheda, Joseph B. Sempa, Elizabeth Tabane, Refuoe Baleni, Tshepang Jiane, Thabiso R. Mofokeng

**Affiliations:** ^1^Department of Internal Medicine, Faculty of Health Sciences, University of the Free State, Bloemfontein, South Africa; ^2^Pharmacovigilance Centre for Public Health Programmes, National Department of Health, Townlands, South Africa; ^3^Department of Biostatistics, Faculty of Health Sciences, University of the Free State, Bloemfontein, South Africa; ^4^Department of Paediatrics and Child Health, Faculty of Health Sciences, University of the Free State, Bloemfontein, South Africa

**Keywords:** pediatric, sub-Saharan Africa, adverse drug reactions, viral load, dolutegravir, ARV-Pediatric Registry, lost to follow-up

## Abstract

Globally, there are approximately 1·5 million children (0–14 years old) living with human immunodeficiency virus/acquired immunodeficiency syndrome (HIV/AIDS), of which 90% are in sub-Saharan Africa. South Africa has the most extensive pediatric HIV care and treatment program. Statistical data from 2023 demonstrated that 152,984 South African children (<15 years) were living with HIV. Despite the remarkable progress toward achieving the UNAIDS 95-95-95 targets in South African adults, the progress in the pediatric population seems to be lagging. HIV/AIDS remains a major factor in the morbidity and mortality of children. Some of the challenges impacting successful treatment outcomes include a high rate of loss to follow-up, virological non-suppression, and difficulties with treatment adherence as the children are reaching adulthood, indicating an urgent need for improved quality of care for children on antiretroviral therapy. The occurrence of adverse drug reactions (ADRs) is one of the problems affecting patient retention in treatment and is associated with an increased risk of incomplete viral suppression. To address these issues, strengthening spontaneous ADR reporting among HIV-infected pediatric patients at healthcare facilities has contributed to developing strategies for the prediction, identification, reporting, and prevention of ADR occurrence. In addition, establishing the ARV-Pediatric Registry has improved the quality of care for children on ART by enabling timely interventions and monitoring the effectiveness and safety of ART. These initiatives will help to address the specific challenges associated with reaching the 95-95-95 targets and provide a pathway forward for sustainable healthcare delivery for this vulnerable population.

## Introduction

Globally, there were approximately 1.5 million children (0–14 years old) living with HIV in 2022, according to the Joint United Nations Program on AIDS (1). Most of the affected children were in the African regions (approximately 92%), primarily in southern Africa (68%) (2). South Africa has the most extensive pediatric HIV care and treatment program. Statistical data from 2023, according to the NAOMI Model district-level HIV, demonstrated that 152,984 South African children (<15 years) were living with HIV (3). Despite the remarkable progress toward achieving the UNAIDS 95-95-95 cascade (which calls for 95% of all People Living with HIV (PLHIV) to know their HIV status, 95% of all people with diagnosed HIV to receive sustained ART, and 95% of all people on ART to have viral suppression) in South African adults, the progress in the pediatric population seems to be lagging as HIV/AIDS remains a major factor in the morbidity and mortality of children (1). Recent data from the National Department of Health (NDoH) (as of October 2024) reported children <15 years at 87–79-72; 87% of children living with HIV know their status, 79% are on treatment, and 72% of those on treatment are virally suppressed. Some of the challenges impacting successful treatment outcomes include a high rate of loss to follow-up, virological non-suppression, challenges with treatment adherence, and treatment failure as the children reach adulthood (4). These indicate an urgent need for improved quality of care for children on antiretroviral therapy. Furthermore, the occurrence of adverse drug reactions (ADRs) is one of the problems affecting patient retention in treatment and is associated with an increased risk of incomplete viral suppression (5). To address these issues, South Africa’s first comprehensive ARV-Pediatric Registry was established in 2019 in the Free State province of South Africa (6). Since the establishment of the registry, a total of 1,034 pediatric patients have been captured on the registry. This initiative is a collaborative project between the NDoH and the University of the Free State. This is to monitor HIV exposed and infected children from birth till the age of 13 years. The registry supports several important activities, including strengthening spontaneous ADR reporting among HIV-infected children, monitoring those switched to a DTG-based regimen, and conducting viral load (VL) monitoring.

The inclusion criteria on the registry include children born to mothers with a documented history of use of ARV drugs during pregnancy, and who also start ARV treatment due to HIV infection. Children without exposure in utero but who are started on ARV therapy due to acquired HIV infection. Children for whom it is not known if there was exposure in utero, but are to be started on ARV therapy due to acquired HIV infection.

## Spontaneous ADR reporting

The spontaneous reporting system is a method of collecting information on suspected ADRs (7). Its main function is to detect new, rare, and serious ADRs that remained undetected in the pre-marketing clinical trials. The system also provides information from real-life clinical practice compared to clinical trials, where children are mostly excluded. Monitoring of ADRs among HIV-infected pediatric patients is a critical public health issue for ART treatment adherence and retention in care.

During the course of establishing the registry, we identified some factors associated with poor ADR reporting, which include:

1. The heavy workload of healthcare professionals with usual clinical activities results in a lack of time to fill in ADR records and follow the outcome of ADRs experienced by the patients.
2. Non-uniformity of knowledge on the pharmacovigilance system among healthcare providers in the hospital.
3. Uncertainty of the ADR diagnosis. Most healthcare providers believe it is necessary to confirm an ADR before reporting; as a result, uncommon ADRs are not reported.
4. The fear of becoming victims of legal liability and possible judicial claims in case of mistakes.

To address the factors associated with poor ADR reporting, exploring interventions that can improve ADR reporting is pertinent, especially among HIV-infected pediatric patients. As part of the registry, we enhanced spontaneous ADR reporting among HIV pediatric patients in the Free State province by engaging trained researchers involved in the ARV-pediatric registry project to complete the ADR forms and conduct patient follow-up. The incidence of ADRs in HIV patients on ARV treatment is a substantial public health problem, especially in the high HIV epidemic countries of sub-Saharan Africa, where health resources are minimal. With the changes in the ART regimen and the complex management of HIV-infected pediatric patients, monitoring and documenting all the suspected ADRs is essential. This intervention has increased the number of ADRs reported and improved the quality of information collected, including the outcome of ADRs experienced among this vulnerable population. Through this intervention, the profile of the most common ADRs experienced by HIV-pediatric patients will be obtained, which will be helpful when screening for possible ADRs associated with the ART regimens.

## Follow-up of children switched to dolutegravir (DTG) according to the new treatment guideline

In 2019, South Africa adopted a DTG-based regimen whereby a new fixed dose combination of Tenofovir (TDF) 300 mg + Lamivudine (3TC) 300 mg + Dolutegravir (DTG) 50 mg (TLD) was recommended for all eligible adults, adolescents, and children over the age of 10 years and weighing 35 kg or more according to the World Health Organization (WHO) guidelines (8). Due to its potency, high barrier to resistance, and tolerability, DTG was recommended for first-line treatment of adults and children with Human Immunodeficiency Virus Type 1 (HIV-1) (8). Following the guidelines of 2018 and 2019 on the use of DTG as first- and second-line treatment for children weighing 35 kg or more, and children weighing at least 20 kg, in 2020, the WHO updated these guidelines recommending the use of dispersible 5 mg formulation of DTG in infants and children living with HIV-1 (9). The DTG-based regimen has been associated with better virological outcomes, including faster viral load suppression compared to protease inhibitors (PI) and non-nucleoside reverse transcriptase inhibitors (NNRTI), as demonstrated by clinical trial reports and a prospective cross-sectional study (9, 10). However, despite the high efficacy of antiretroviral treatment, no medication is free from adverse reactions, and DTG is no exception. The DTG-based regimen has been linked with neuropsychiatric manifestations (including psychiatric events and suicidal ideation or behavior) and sleep disturbances in children and adolescents in a recent clinical trial (11). Another concern regarding the switch of all pediatric patients to a DTG-based regimen is the possibility of potential drug interaction, especially in the South African context, given the high rates of HIV and tuberculosis co-infection. For instance, information about possible interaction between rifampicin and DTG-based regimens is critical to this cohort. Opinion differs regarding the necessity of doubling the dose of dolutegravir to 50 mg twice daily according to the current WHO guidelines. Also, there has been a recent report of drug resistance to DTG in a pediatric South African patient (12). The ARV-Pediatric Registry has helped collect central data on baseline characteristics of pediatric patients at the initiation of antiretroviral therapy and following up as the patients are switched to DTG-based regimens. Thus, the registry serves as a valuable data source for monitoring the safety and effectiveness of DTG-based ART for pediatric patients, considering the recent call to switch all pediatric patients on ART to DTG-based ART in South Africa. This registry will enhance the documentation of suspected adverse drug reactions that may be associated with DTG-based regimens and potential drug interactions, as the registry documents information on co-morbidities. According to the registry, out of 1,034 patients, 57 patients (36 females, 21 males) have been switched to a DTG-based regimen.

## Viral load monitoring

Viral load (VL) monitoring is an important aspect of antiretroviral therapy service delivery (13). VL non-suppression poses a challenge to achieving the UNAIDS 95-95-95 targets. The registry provides information on the baseline and follow-up viral load values after ART initiation. A viral load > 1,000 implies severe or uncontrolled infection (6). During the follow-up period, a persistent high viral load (> 1,000 copies) requires the patient’s re-evaluation and ART regimen switch. Out of 1,034 patients captured on the registry, baseline VL and follow-up VL monitoring have been conducted for 845 patients. The VL monitoring will help to reduce the development of drug resistance, with the ultimate goal of viral suppression (13). It is not just enough to simply initiate ART for HIV-infected children. Regular therapeutic monitoring, treatment failure prevention initiatives, and ART resistance testing should also be prioritized (4). Despite the high infection rates of HIV among children in sub-Saharan Africa, only a few studies have explored issues relating to pediatric ART initiation and sustainability (4). In a recent report on the global estimates of viral suppression in children and adolescents, evidence has shown that progress toward approaching the global target of 95% viral suppression in this vulnerable group is much slower. Therefore, Substantial efforts are needed to reach the viral suppression target for children and adolescents (14).

## Lost to follow-up (LTFU) rate and outcomes

In recent global data, an increase in mortality rate estimates accounting for worse outcomes among lost to follow-up has been reported in tracing data, which is higher than mortality estimates from routine care settings in African regions (2). In line with the recent rollout by the Minister of Health, contact tracking of lost to follow-up patients is an integral part of patient retention in care (15). Because LTFU data is not part of the routine data captured on the registry, it is difficult to get accurate data on the rate and outcomes. We noticed inconsistencies in the lost to follow-up report, as many patients who came for medication refills were not captured for clinic visits. Thus, most patients who are up to date regarding follow-up appointments and medication refills were captured as lost to follow-up. In February 2025, a new contact-tracking register was introduced to the clinics to record the details and outcomes of the contact-tracking (sample template attached as supplementary material). Due to the importance of lost to follow-up data and their contribution to mortality rate estimates, the registry team is currently working with the clinics for up-to-date information on lost to follow-up data and detailed outcomes of the contact-tracking process.

## Discussion

Some of the factors that could help to address the specific challenges associated with reaching the 95-95-95 targets and provide a pathway forward for sustainable healthcare delivery for this vulnerable population include spontaneous ADR reporting, real-time follow-up of children on ART regimen switch (especially new regimens), VL monitoring and reducing dropout rates via efficient contact-tracking systems lost to follow-up patients. Despite the effectiveness of spontaneous ADR reporting in managing ADRs to ART, a literature review has shown that most reports of ADRs are retrospectively reported from patient files (16, 17), with a few reports using active pharmacovigilance (18). For adequate monitoring of previously reported ADRs and detection of new ones, we propose that spontaneous ADR reporting or active surveillance should be included in pediatric patient care. This will facilitate patient retention in care and contribute to developing strategies for predicting, identifying, reporting, and preventing ADR occurrence.

As children are switched to DTG-based regimens according to the new treatment guidelines, a real-time follow-up on treatment is important to identify new ADRs that may be associated with the regimen. With the recent report of psychiatric events and sleep disturbances reported in children and adolescents using DTG-based regimens (11), it is important to institute a mechanism of long-term follow-up on treatment for the utmost management of adverse events. Evidence of possible interaction between rifampicin and DTG-based regimens is critical to this cohort. In a recent report, twice-daily dolutegravir with rifampicin in children (age range 5–13 years) is well tolerated without adverse events, results in viral suppression, and achieves adequate plasma drug concentrations (19). However, this study could not confirm the efficacy of once-daily dolutegravir during rifampicin co-administration in children. Report from a clinical trial study which suggested that twice-daily DTG might be unnecessary in people with HIV on rifampicin-based antituberculosis therapy, used participants older than 18 years of age (20). It is uncertain whether children under 14 will react the same way. This is in contrast to the WHO guidelines of twice-daily dolutegravir for people with HIV on rifampicin-based antituberculosis therapy in an attempt to address the issue of medication out of stock in high-burden countries. Therefore, clinical data is required to prove this theory, similar to the adult population.

Viral load monitoring in children is an important indicator for assessing treatment efficacy. Regarding viral load monitoring in South Africa, as of October 2024, the progress on the 95-95-95 National HIV Treatment Cascade in children (<15) shows that out of 79% that are on treatment, 72% are virally suppressed (NDoH data). Also, our retrospective data collection for the registry shows optimal VL testing, as 82% of the patients have done their baseline and follow-up VL monitoring. In contrast, in a recent review, authors have reported that VL monitoring is suboptimal in many sub-Saharan African countries; thus, health system strengthening is required for the sustainability of HIV treatment programs and the achievement of 95-95-95 targets (10). To achieve VL suppression, children must be retained in treatment. Lost to follow-up from ART treatment is a global challenge, especially in developing countries (21). Monitoring lost to follow-up data will contribute to patient retention in care as part of a commitment to ensure South Africa reaches their target. Also, continuous monitoring of patients captured in the ARV-Pediatric Registry will help keep more patients on first-line ART regimens longer, avoiding the more expensive and dangerous second-line ART regimens.

In conclusion, the ultimate goal of establishing the ARV-Pediatric Registry is to institute a safety monitoring mechanism that is helpful to collect central data on baseline characteristics of HIV positive children at the initiation of antiretroviral therapy and follow-up on treatment. The registry is a vital initiative that will significantly improve the quality of care for children on ART treatment by enabling timely interventions and monitoring the effectiveness and safety of ART. This initiative will help to address the specific challenges associated with reaching the 95-95-95 targets and provide a pathway forward for sustainable healthcare delivery for this vulnerable population.

## Data Availability

The raw data supporting the conclusions of this article will be made available by the authors, without undue reservation.
